# Hypoxic blackout in dynamic apnea: A case report

**DOI:** 10.1016/j.jphyss.2026.100060

**Published:** 2026-01-26

**Authors:** Eric Mulder, Isak Löfquist, Felix Schagatay, Arne Sieber, Erika Schagatay

**Affiliations:** a Department of Health Sciences, Mid Sweden University, Sweden; b Internal and Acute Medicine, Karolinska University Hospital, Solna, Sweden; c Oxygen Scientific GmhB, Graz, Austria; d Swedish Winter Sport Research Centre, Mid Sweden University, Östersund, Sweden

**Keywords:** Freediving, Breath-holding, Loss of consciousness, Hypoxia, Safety

## Abstract

Blackout (BO) in breath-hold diving is attributed to cerebral hypoxia, yet direct observations are rare. We continuously recorded arterial oxygen saturation (SpO₂) and heart rate (HR) in 11 trained freedivers (5 females) performing two dynamic apneas (75 m, 100 m) using a waterproof forehead oximeter. One diver experienced BO at the end of a 100 m dive (SpO₂ 51 %), recovering within 5 s. Group SpO₂ fell from 98 ± 1 % to 77 ± 9 % (75 m) and 68 ± 9 % (100 m; range 51–83 %), while mean HR declined from 83 ± 12 to 43 ± 8 and 40 ± 4bpm, respectively. No arrhythmias were detected. Within-diver SpO₂ nadirs were consistent between distances (r = 0.93), whereas HR nadirs were not (r = 0.40). This case confirms BO can occur at SpO₂ values around 50 %, even in the absence of arrhythmia. The BO diver consistently showed the lowest SpO₂, indicating profound hypoxemia as the most likely contributing factor. Findings support individualized risk screening based on early desaturation patterns in submaximal dives.

## Highlights

• Continuous underwater SpO₂–HR monitoring captured a real blackout event.
• Blackout occurred at 51 % SpO₂ without any detectable arrhythmia.
• SpO₂ nadirs were highly consistent within divers across two distances.
• Blackout diver showed fastest and deepest desaturation at both distances.
• Early desaturation patterns may help identify individual blackout risk.

## Introduction

Breath-hold diving (freediving) is practiced as a profession to acquire underwater resources, as a recreational activity, and in competitive sport. Competitive freediving exposes the human body to pronounced physiological stress, including profound hypoxemia despite preventive cardiovascular adjustments of the diving response. One of the most serious complications is loss of consciousness, or blackout, which in aquatic environments may lead to drowning [1].

Although blackout-related deaths appear to be most common in recreational divers [2], blackouts occur most frequently during competition or preparatory training [3]. In these settings, rigorous safety protocols, including the presence of safety divers, help to mitigate the risk of drowning [4]. This makes competitive freediving a suitable model for investigating the causes of blackout.

Blackout has traditionally been attributed to severe brain hypoxia, but recent studies suggest that susceptibility is highly individual, and the influence of additional physiological triggers remain incompletely understood [5], [6], [7]. Blackout is often assumed to occur when arterial oxygen saturation (SpO_2_) approaches ∼50 % [8]. However, many trained freedivers have been shown to sustain lower SpO_2_ levels without losing consciousness (38 % by Ferretti and colleagues [9]; 25 % by McKnight and colleagues [10]; 44 % by Mulder and colleagues [11]; 31 % by Valdivia-Valdivia and colleagues [12]). This suggests that tolerated SpO₂ thresholds for sustained brain function are highly individual, or that other factors contribute to blackout susceptibility.

In a recent case study, we reported a blackout during a static apnea competition that coincided with a bigeminal arrhythmia pattern, despite an SpO_2_ of 57 %, i.e., above the level typically associated with blackout, suggesting a potential contribution of cardiac dysfunction [7]. In that study, we speculated that hypoxia may first affect cardiac function, which would in turn compromise cerebral perfusion and indirectly cause brain hypoxia.

In the present study, we monitored a group of trained freedivers performing two dynamic apneas, where divers swim horizontally underwater, as part of a standardized testing protocol designed to assess freediving performance. Notably, one diver experienced a blackout at the conclusion of a 100 m attempt. This case provided a rare opportunity to examine the immediate physiological state preceding blackout and to compare the event with responses of non-blackout divers under the same conditions. The aim of the current study was therefore to: (1) describe SpO_2_ and heart rate patterns preceding a blackout in dynamic apnea, and (2) evaluate whether identifiable physiological predictors, such as oxygen desaturation levels, arrhythmia or degree of bradycardia, differentiate blackout from non-blackout dives.

## Methods

### Participants

Eleven trained freedivers (5 females, mean ± SD age: 35 ± 6 years, height: 178 ± 10 cm, body mass: 71 ± 13 kg) volunteered to participate. All participants were active freedivers with a minimum of two years of regular training. Mean ± SD personal best in dynamic apnea was 142 ± 58 m. The study protocol was approved by the Swedish Research Ethics Authority (Dnr 2024–07532–01), conformed to the Declaration of Helsinki with the exemption of database preregistration, and all participants provided written informed consent prior to participation.

### Equipment

SpO₂ and heart rate were continuously recorded using a prototype water- and pressure-proof pulse oximeter [13]. The device consisted of a reflective pulse oximetry sensor applied on the diver’s forehead under the wetsuit hood, connected by cable to a datalogger secured inside the wetsuit on the diver’s back. A chest strap (Polar T-34, Polar Electro Oy, Kemple, Finland) around the thorax transmitted R-R intervals wirelessly to the same data logger. SpO₂ and heart rate data were sampled at 50 Hz.

### Procedures

A 75 m followed by a 100 m dynamic apnea with bifins were performed in a 25 m outdoor pool, each preceded by 5 min of immersed rest. Freedivers used personal equipment and routines but, for standardization, were instructed to avoid pre-dive lung packing [14] and post-dive hook breathing [15]. Dives were performed with bifins, noseclip, weights, and a hooded wetsuit. Attempts were terminated voluntarily if the planned distance could not be reached. Each dive was accompanied by an experienced safety diver. Some divers performed brief push-offs after immersion to adjust weighting. All participants were monitored ≥ 5 min post-protocol. Ambient air temperature was 30 ± 4 °C and water temperature 24 ± 1 °C.

### Analysis

Data from the diver who experienced a blackout were compared with non-blackout divers. Descriptive statistics are reported as mean ± SD unless otherwise stated. Signal quality was reviewed post hoc; periods with artifacts or signal loss ≥ 10 s were excluded from analysis. Data quality was insufficient for full analysis in two divers; SpO_2_ analyses therefore included nine divers (five females). One additional diver had incomplete heart rate data; heart rate analyses included eight divers.

#### SpO2 and heart rate processing

Photoplethysmographic (PPG) signals were corrected for light-intensity steps, detrended (2 s moving window), and band-pass filtered to reduce noise. R-values were computed with a root-mean-square (RMS) method before converting to SpO₂ via device-specific calibration constants. The mean of the three lowest consecutive SpO₂ values occurring within 20 s after surfacing was taken as the nadir, which, due to the circulatory delay, corresponded with the end of each dive. Nadir heart rate was the lowest 5 s average detected during the dive [7]. Baseline SpO₂ and heart rate were computed over a 60 s stable period, from 3 until 2 min pre-dive, before the 2 min countdown started.

#### Arrhythmia screening

Beat-to-beat R–R intervals (ms; column 15 in each file) were analyzed to screen for potential arrhythmic events using established RR-based approaches [16]. For each dive, Poincaré plots (RR_n_ vs RR_n+1_) and difference plots (dRR_n_ vs dRR_n+1_) were computed to assess rhythm geometry, together with its derived non-linear measurements SD1 and SD2. Irregularity was quantified by sample entropy (m = 2, r = 0.2·SD), while alternating short–long patterns were evaluated by an autocorrelation-based bigeminy score (lag-1, lag-2, and beat-to-beat alternation). The RR–dRR (RdR) map was generated using 25 ms bins, and the number of non-empty cells (NEC) was determined in 64-beat sliding windows [17]. These complementary metrics allow discrimination between normal sinus rhythm, bigeminy, and atrial fibrillation based solely on RR dynamics [16], [18].

#### Correlations

Normality of variables included in the correlation analyses was assessed using the Shapiro–Wilk test. No significant deviations from normality were observed; therefore, Pearson correlations were computed to assess (a) within-diver consistency of nadirs between 75 m vs 100 m for SpO₂ and heart rate, and (b) associations between heart rate reduction (%) and SpO₂ desaturation (%) at each distance. Given sample size, these were descriptive (including CIs).

## Results

All 11 divers completed the intended 75 and 100 m distances. One participant experienced a brief blackout at the end of the 100 m dive. The incident was managed immediately by the safety diver using the “blow-tap-talk” method [19]. The diver regained consciousness within 5 s, and no residual symptoms were reported at 10 min after recovery. No other adverse events occurred during the dives. Mean dive duration for the 75 m dive was 88 ± 6 s, and 114 ± 16 s for the 100 m dive. The diver who experienced blackout completed the 100 m dive in 128 s.

The diver who experienced blackout did not differ markedly from the other participants in basic physical characteristics or training background. This diver was 26 years old, 176 cm tall, with a body mass of 64 kg, 1.5 years of freediving training, and a self-reported training volume of 8 h/week during the two months preceding the study. These values fell within the range observed across the group (age: 26 – 42 years; height: 158 – 191 cm; body mass: 49 – 89 kg; training experience: 1.5 – 16 years; recent training volume: 0 – 20 h/week). The diver’s dynamic apnea personal best (100 m) also fell within the range of the group (75 – 253 m).

### Arterial oxygen saturation

**Fig. 1:**
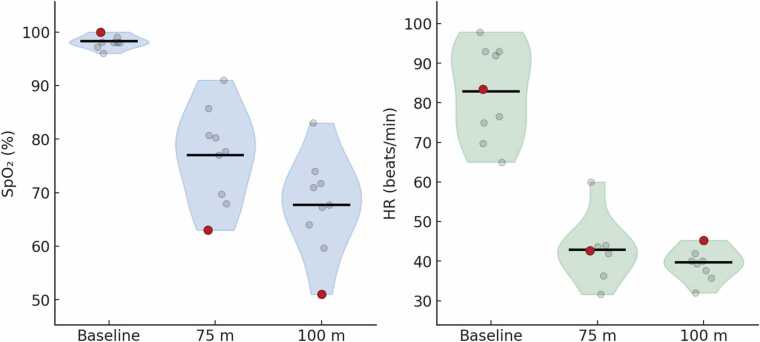
Arterial oxygen saturation (SpO₂) and heart rate (HR) responses in trained freedivers during 75 m and 100 m dynamic apnea with bifins (DYN). Each violin represents the distribution of individual nadir values, with the horizontal black bars indicating group means. Individual diver values are shown as grey circles. The red filled circle marks the diver who experienced a blackout at the end of the 100 m dive, who also exhibited the lowest SpO₂ during the 75 m dive.

Mean SpO_2_ (n = 9) decreased from a baseline of 98 ± 1 % to a nadir of 77 ± 9 % (range: 63 – 91 %) after the 75 m dive – a mean reduction by 21.6 ± 8.7 %. During the 100 m dives, mean nadir SpO_2_ reached 68 ± 9 % (range: 51 – 83 %), corresponding to a mean reduction by 31.1 ± 9.1 %. The diver with blackout exhibited the lowest SpO₂ (51 %) among all divers at 100 m (Fig. 1). This diver also had the lowest SpO_2_ after the 75 m dive (63 %). Despite the small group size (n = 9), there was an association between individual SpO_2_ responses during the 75 m dive and the 100 m dive (r = 0.93; p < 0.01), whereas heart rate nadirs were not associated (r = 0.40). Neither was there any association between individual heart rate reduction and SpO₂ desaturation. SpO₂ nadirs generally occurred just after surfacing, consistent with a circulatory cardiopulmonary transit delay.

### Heart rate

**Fig. 2:**
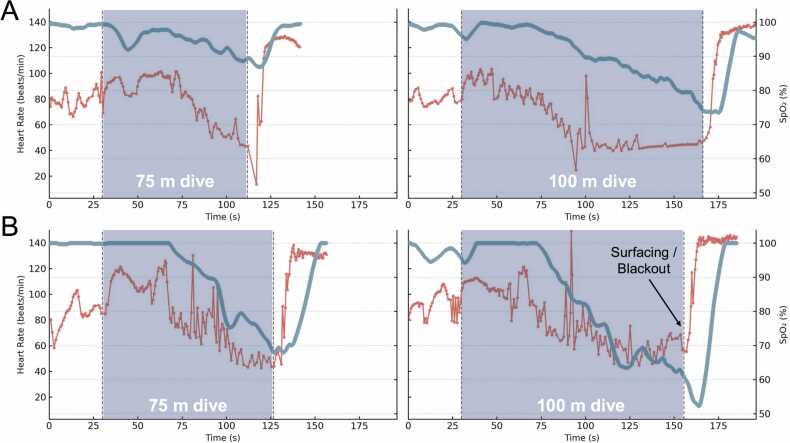
Continuous recordings of heart rate and arterial oxygen saturation (SpO₂) during 75 m and 100 m dynamic apnea in two trained freedivers. Diver 1 (A) represents a non-blackout control diver, and Diver 2 (B) the participant who experienced a brief blackout immediately after the 100 m dive; the moment of surfacing coinciding with the blackout is indicated by a black arrow. Vertical dashed lines indicate dive start and end; each panel includes 30 s pre- and post-dive. Heart rate (red) is derived from R-R intervals and plotted on the left axis (beats/min), and SpO₂ (blue) is plotted on the right axis (%). Note the progressive diving bradycardia and desaturation throughout the dives, with a more rapid and pronounced SpO₂ decline in Diver 2, culminating in a nadir of ≈ 51 % after surfacing.

Mean heart rate (n = 8) dropped from 83 ± 12 beats/min at baseline to a nadir of 43 ± 8 beats/min during the 75 m dive (a 46.5 ± 13.0 % reduction), and to 40 ± 4 beats/min during the 100 m dive (a 51.4 ± 7.0 % reduction; Fig. 1). Heart rate responses of the diver who blacked out remained within the group range, with a nadir of 43 beats/min (49 % reduction) during the 75 m dive and 45 beats/min (46 % reduction) during the 100 m dive. The heart rate nadirs generally occurred near or at the end of the dives (Fig. 2).

### Arrhythmia screening

The diver who blacked out exhibited no arrhythmic features. Across all participants (n = 8) and both dynamic apnea distances (75 and 100 m), RR-based analyses found no evidence of sustained arrhythmia. Poincaré plots displayed single, elongated clusters consistent with normal sinus modulation, without the bifurcated or scattered geometries typical of bigeminy or atrial fibrillation (Supplemental material S1). Quantitative Poincaré plot analysis showed that SD1, SD2, and the SD1/SD2 ratio in the diver who experienced blackout fell within the range observed in the remaining divers. Specifically, SD1 was 142 ms, SD2 was 339 ms, and the SD1/SD2 ratio was 0.42 in the diver who blacked out, compared with group values of 174 ± 59 ms for SD1, 388 ± 100 ms for SD2, and an SD1/SD2 ratio of 0.51 ± 0.33. Sample entropy values were uniformly low to moderate (0.08–0.69), indicating structured rather than random variability. Bigeminy scores remained below 0.5 in all cases, and lag-1 autocorrelations were strongly positive, excluding alternating short–long RR patterns. RdR non-empty cell counts (64-beat windows) occasionally exceeded 40 cells but without associated morphological irregularity, consistent with normal respiratory or autonomic fluctuations during diving. To further examine cardiac rhythm at the time of the event, the R–R interval time series spanning −30 to + 30 s relative to surfacing during the blackout dive is shown in Supplemental material S2.

### Relationships between SpO_2_ and heart rate

Nadir SpO₂ at 75 m was strongly associated with nadir SpO₂ at 100 m (r = 0.93, 95 % CI 0.69–0.99; n = 9), indicating a high degree of individual consistency in oxygen desaturation across both dives. In contrast, nadir heart rate showed only a modest and imprecise association between 75 and 100 m (r = 0.40, 95 % CI −0.42–0.86; n = 8).

The association between maximum heart rate reduction (%) and SpO₂ desaturation (percentage-point drop) was small-to-moderate and statistically imprecise at both 75 m (r = 0.47, 95 % CI −0.35–0.88; n = 8) and 100 m (r = 0.31, 95 % CI −0.44–0.81; n = 9), suggesting that the magnitude of bradycardia alone did not reliably predict the magnitude of oxygen desaturation.

## Discussion

Continuous monitoring of SpO_2_ and heart rate using the prototype pulse oximeter enabled us to capture these key variables throughout a dynamic apnea in which a blackout occurred. The diver who lost consciousness exhibited the lowest SpO_2_ of all participants, reaching 51 % at the end of the 100 m dive. The same diver also showed the lowest SpO_2_ during the preceding 75 m dive (63 %), which did not result in blackout. For this individual, the limit for consciousness therefore appears to lie between 51 % and 63 % SpO_2_.

During the 100 m dive, the heart rate of the diver who blacked out remained free from arrhythmia, and the nadir heart rate of 45 beats/min was comparable to that of the other divers. These findings suggest that the blackout was most consistent with severe arterial oxygen desaturation rather than detectable cardiac dysfunction. This supports the long-standing assumption that brain hypoxemia alone can cause loss of consciousness in freedivers – a relationship that has often been suggested but, until now, never documented through continuous physiological recordings during an actual diving event.

We previously reported a blackout during a maximal static apnea [7], where heart rate patterns revealed bigeminal arrhythmia preceding loss of consciousness. In that case, the diver’s SpO_2_ at blackout (57 %) was similar to that of control divers who did not lose consciousness and notably higher than the SpO_2_ associated with blackout in the current study. This comparison suggests that blackout mechanisms may differ between divers and conditions: in some cases, cardiac rhythm disturbances may compromise stroke volume and cerebral perfusion, whereas in others – as in the present dynamic apnea – blackout may result directly from cerebral hypoxia despite preserved cardiac function [20]. Across the eight divers with analyzable heart rate, standardized Poincaré and RR-interval irregularity analyses showed no sustained bigeminy or other overt arrhythmic patterns, further supporting that the current blackout was not precipitated by arrhythmia.

Previous studies have proposed that simultaneous activation of sympathetic and parasympathetic pathways may cause an “autonomic conflict”, potentially increasing the risk of arrhythmias under certain circumstances [21], [22]. Indeed, autonomic imbalance has been implicated in both clinical and diving-related arrhythmic events [6], [23], [24]. However, despite moderate bradycardia, no arrhythmia was observed in the present blackout case, supporting the interpretation that severe hypoxemia was a likely contributing factor in this case.

Our results confirm that blackout may occur when SpO_2_ approaches ∼50 % [8] in trained freedivers. However, other divers have been shown to sustain consciousness at SpO_2_ levels as low as 25 – 44 % [9], [10], [11], [12], indicating substantial individual variability in hypoxia tolerance.

No clear relationship was found between the magnitude of bradycardia and the degree of oxygen desaturation at any diving distance [25], [26], although the small sample size (n = 8) limits this conclusion. The diver who blacked out exhibited both a typical heart rate reduction associated with the diving response and a rapid, severe oxygen desaturation. Individual differences in desaturation rate beyond those explained by the diving response may reflect variability in oxygen storage capacity – such as hemoglobin concentration, splenic contraction, and muscle myoglobin content – which merits further investigation to identify individual physiological markers of diving capacity. It is also notable that the diver who experienced blackout had the lowest training experience and one of the lowest dynamic apnea personal best in the group. While these factors cannot be interpreted causally, they may reflect differences in physiological adaptation to repeated hypoxic exposure, pacing strategies, or tolerance to hypoxemia, and should be considered contextual rather than mechanistic contributors in this case.

The finding that the diver who blacked out already displayed marked oxygen desaturation after the 75 m dive may have practical implications for safety monitoring and individual risk assessment in freediving. Early oxygen desaturation patterns during shorter dives could potentially serve as individualized predictors of blackout risk in longer or more demanding efforts; however, this observation should be considered hypothesis-generating and requires confirmation in larger datasets. Although continuous underwater monitoring systems are not yet commonly available in training or competition settings, our results highlight their potential for identifying personal physiological limits and preventing severe hypoxemia or blackout.

The generalizability of these findings is limited by the single blackout event and the modest sample size. Larger datasets including multiple blackout observations will be required to establish SpO₂ thresholds and document other potentially contributing factors. While blackout is hazardous in unsupervised diving, it can be studied safely in controlled environments, such as pool competitions or supervised training sessions with safety divers present.

## Conclusions

The present study offers rare documentation of continuous SpO_2_ and heart rate patterns across a blackout event during freediving, demonstrating that loss of consciousness may occur without cardiac rhythm disturbance. This case shows that blackout can occur at SpO_2_ values around 50 %, which are higher than those previously observed in conscious freedivers, suggesting that hypoxia tolerance varies substantially between individuals. The diver who blacked out exhibited the lowest SpO_2_ among all participants, supporting the interpretation that arterial oxygen desaturation may be a critical determinant of blackout risk in this case. The observation that this diver also showed pronounced oxygen desaturation after the shorter 75 m dive suggests that early or rapid desaturation patterns could serve as practical indicators for risk screening and individualized safety strategies in freediving.

## CRediT authorship contribution statement

**Eric Mulder:** Writing – review & editing, Writing – original draft, Visualization, Formal analysis. **Isak Löfquist:** Writing – review & editing, Methodology, Investigation. **Felix Schagatay:** Writing – review & editing, Investigation. **Arne Sieber:** Writing – review & editing, Resources, Methodology. **Erika Schagatay:** Writing – review & editing, Writing – original draft, Project administration, Methodology, Investigation, Funding acquisition, Data curation, Conceptualization.

## Ethics approval and consent to participate

This study was approved by the Swedish Ethical Review Authority (Dnr 2024–07532–01) and conducted in accordance with the Declaration of Helsinki. All participants received written and verbal information and provided written informed consent prior to participation.

## Consent for publication

All participants provided consent for publication of anonymized data. No identifiable personal information is included in this manuscript.

## Funding

This work was supported by the Francis family. The funders had no role in study design, data collection, analysis, interpretation, or writing of the manuscript.

## Declaration of Competing Interest

The authors declare the following financial interests/personal relationships which may be considered as potential competing interests: Eric Mulder reports financial support was provided by Francis Family. If there are other authors, they declare that they have no known competing financial interests or personal relationships that could have appeared to influence the work reported in this paper.

## Data Availability

The datasets generated and analysed during the current study are not publicly available due to restrictions imposed by the ethical approval, which prohibits sharing of physiological data that may allow re-identification of individual divers.

## Glossary

Apnea: Voluntary breath-holding, performed underwater or on land.
Blackout: Loss of consciousness occurring during or immediately after apnea.
Bradycardia: A reduction in heart rate below resting levels; in freediving, part of the diving response.
Diving Response: Reflexive physiological response to apnea and facial immersion, including bradycardia and peripheral vasoconstriction.
Dynamic Apnea: Competitive freediving discipline in which divers swim horizontally underwater for maximum distance using – in this case – bifins.
Hypoxemia: Low arterial oxygen content, measured here as reduced SpO₂.
Nadir SpO₂ / Nadir HR: The lowest recorded oxygen saturation or heart rate during or immediately after a dive.
Pulse Oximetry (SpO₂): Non-invasive optical method estimating arterial oxygen saturation.
R–R Interval (RR): Time between consecutive heartbeats, used for calculating heart rate and detecting arrhythmias.
Poincaré Plot: Graphical representation of RR intervals used to assess beat-to-beat heart rhythm variability.
Safety Diver: Trained individual responsible for monitoring and assisting freedivers during underwater activities.

## Appendix A Supplementary material

**Supplemental material S1.** Poincaré plots (RR_*n*_ vs. RR_*n+1*_) for all participants during the 100 m dynamic apnea. Each panel represents one diver. The plots illustrate beat-to-beat heart rate dynamics and were used to screen for sustained arrhythmic patterns

**Supplemental material S2.** R–R interval time series recorded from −30 to + 30 s relative to surfacing during the 100 m dynamic apnea in the diver who experienced blackout. The trace shows no evidence of abrupt rhythm irregularity or alternating short–long patterns around the time of loss of consciousness
