# Supplementary figures and images for: Hypoxic blackout in dynamic apnea: A case report
Source: J Physiol Sci. 2026 Jan 26;76(1):100060. doi: 10.1016/j.jphyss.2026.100060 (PMC12874292; doi:10.1016/j.jphyss.2026.100060)

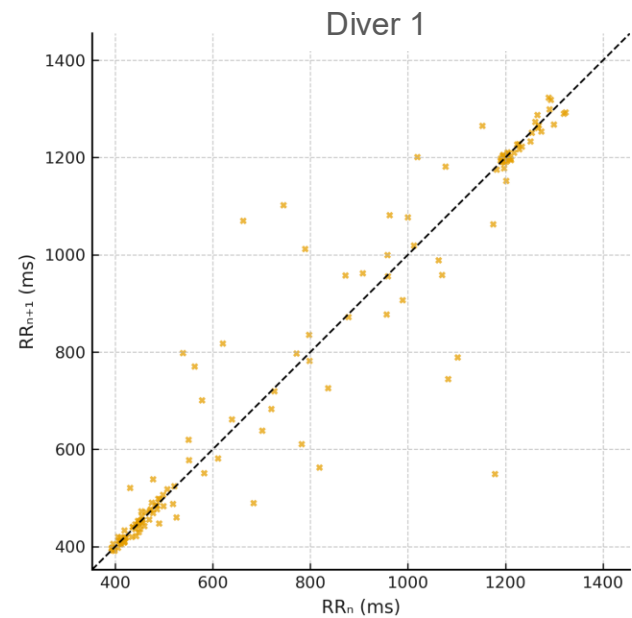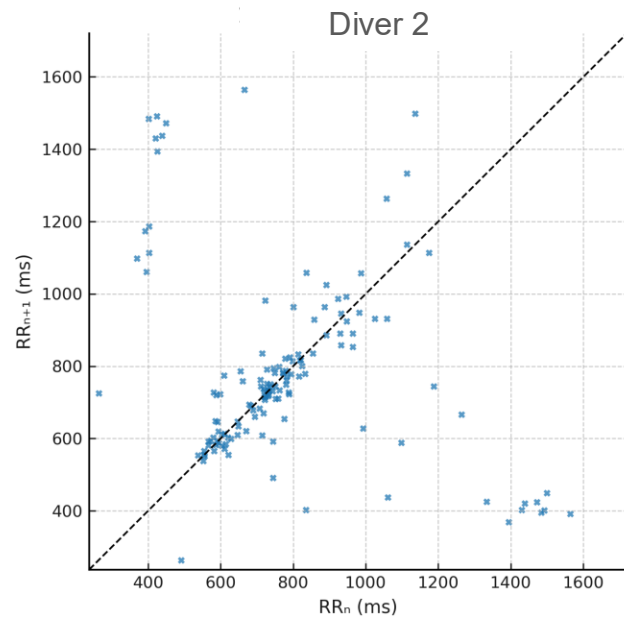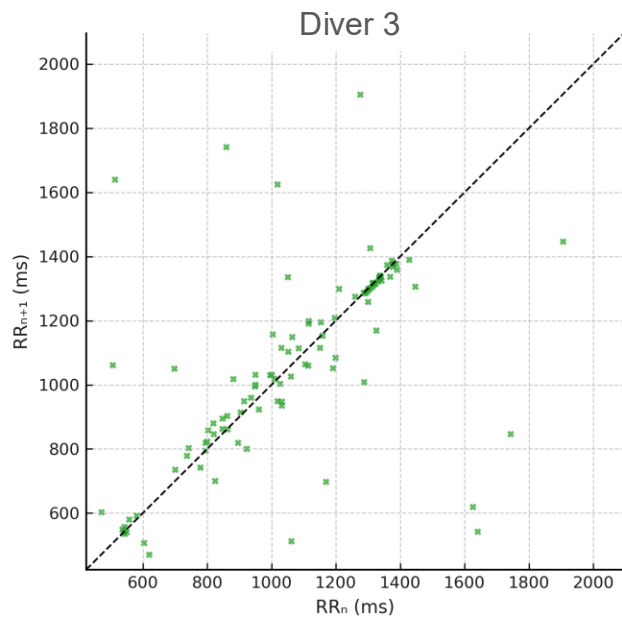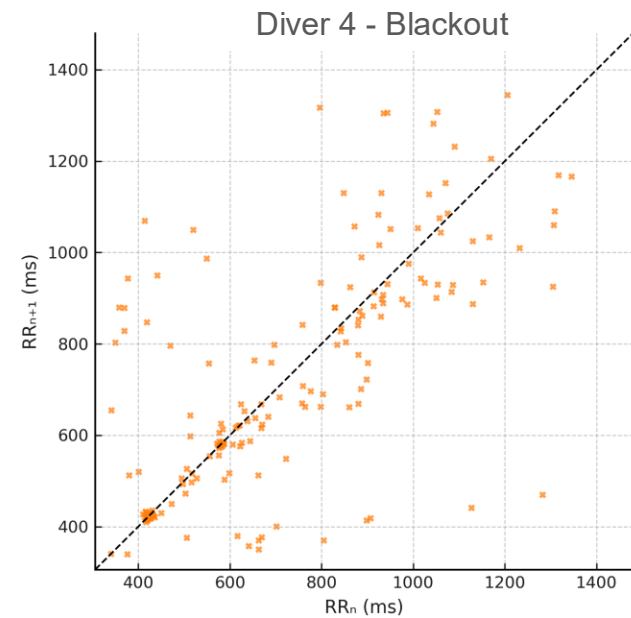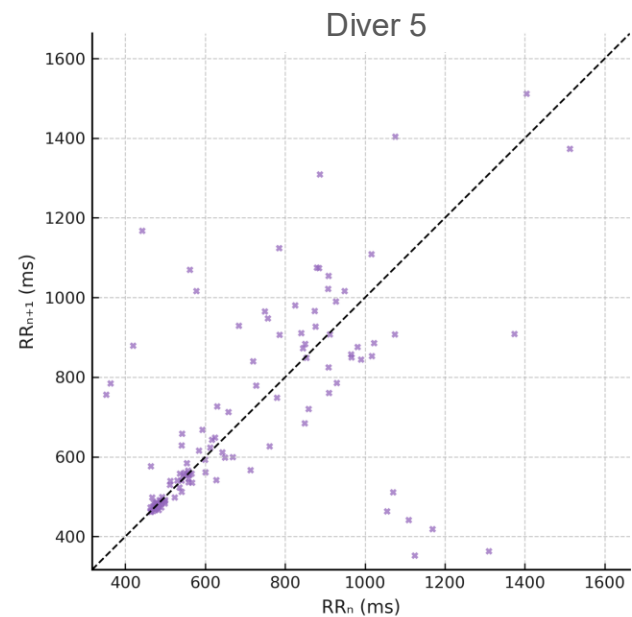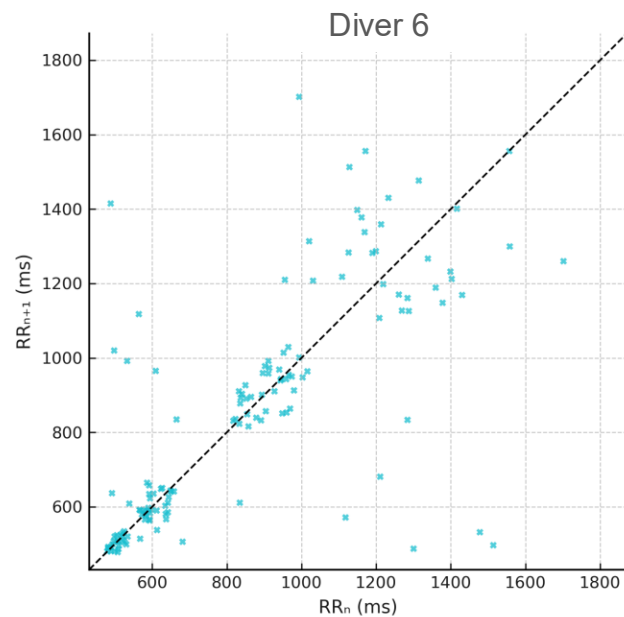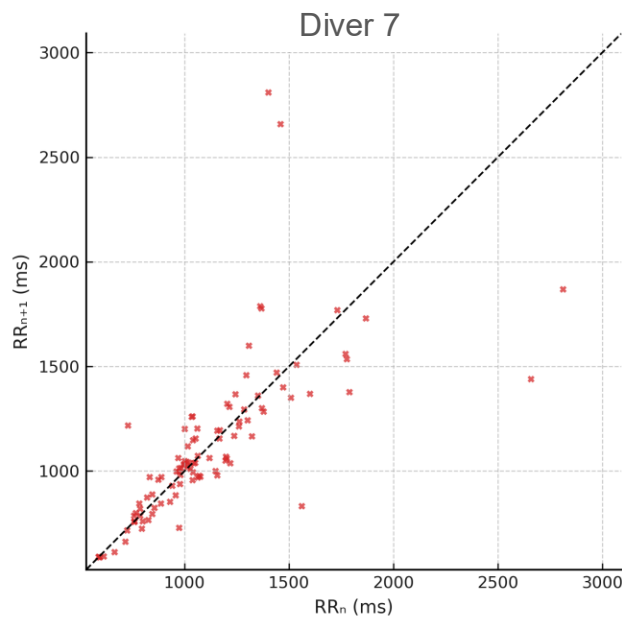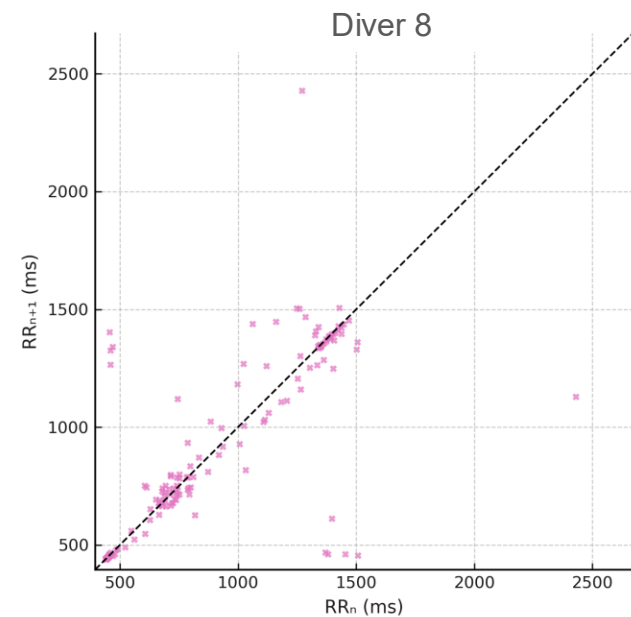

Supplement: Supplementary file 1 — Supplemental material S1. Poincaré plots (RRn vs. RRn+1) for all participants during the 100 m dynamic apnea. Each panel represents one diver. The plots illustrate beat-to-beat heart rate dynamics and were used to screen for sustained arrhythmic patterns [file mmc1.pdf]

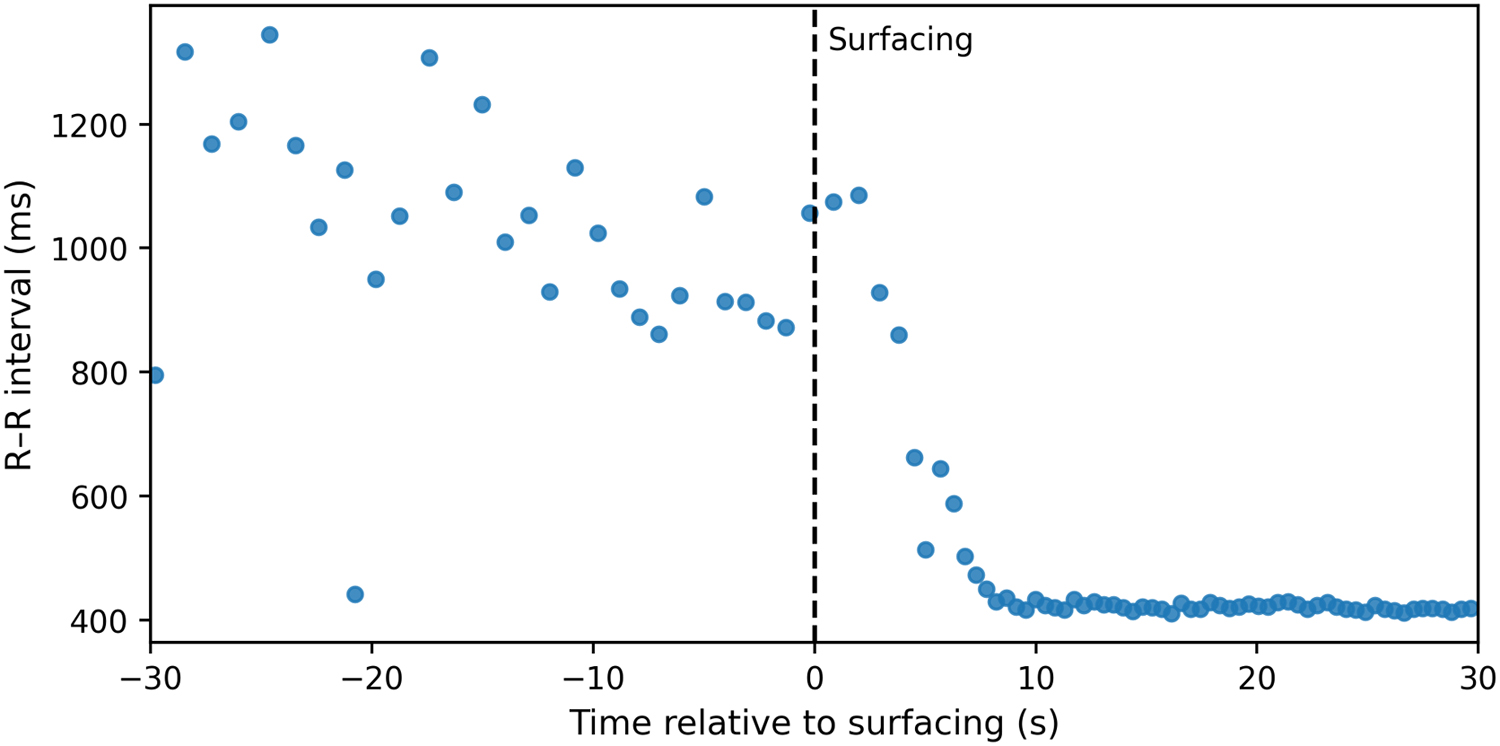

Supplement: Supplementary file 2 — Supplemental material S2. R–R interval time series recorded from −30 to + 30 s relative to surfacing during the 100 m dynamic apnea in the diver who experienced blackout. The trace shows no evidence of abrupt rhythm irregularity or alternating short–long patterns around the time of loss of consciousness [file mmc2.jpg]
